# Causes, Solutions and Health Inequalities: Comparing Perspectives of Professional Stakeholders and Community Participants Experiencing Low Income and Poor Health in London

**DOI:** 10.1111/hex.70128

**Published:** 2024-12-17

**Authors:** Neil McHugh, Rachel Baker, Cam Donaldson, Ahalya Bala, Marta Mojarrieta, Gregory White, Olga Biosca

**Affiliations:** ^1^ Yunus Centre for Social Business and Health, Glasgow Caledonian University Glasgow Scotland UK; ^2^ National Centre for Epidemiology & Population Health (NCEPH), Australian National University Canberra Australia; ^3^ School of Law and Social Sciences Oxford Brookes University Oxford UK; ^4^ National Centre for Social Research London UK

**Keywords:** causes, health inequalities, lay perceptions, Q methodology, solutions

## Abstract

**Background:**

Engaging with the public can influence policy decisions, particularly towards more radical policy change. While established research exists exploring public perceptions on causes of health inequalities, much less exists on how to tackle health inequalities in the UK. Despite an emphasis on ‘lived experience’, currently no study has focused on how individuals with very poor health conceive of both causes of, and solutions to, health inequalities.

**Methods:**

Q methodology was used to identify and describe the shared perspectives that exist on causes of, and solutions to, health inequalities experienced in low‐income communities. Community participants living with low‐incomes and poor health (*n* = 20) and professional stakeholders (*n* = 20) from London rank ordered 34 ‘Causes’ and 39 ‘Solutions’ statements onto quasi‐normal shaped grids according to their point of view. Factor analysis defined factors for both ‘Causes’ and ‘Solutions’.

**Results:**

Analysis produced three‐factor solutions for both the ‘Causes’ and ‘Solutions’. ‘Causes’ are (i) ‘Systemic inequality and poverty’, (ii) ‘Ignored and marginalised communities’, (iii) ‘Precariousness, chronic stress and hopelessness’. ‘Solutions’ are (i) ‘Meeting basic needs and providing opportunities to thrive’, (ii) ‘Empowering individuals to take control’, (iii) ‘Supporting healthy choices’. No professional stakeholders aligned with ‘Ignored and marginalised communities’ while at least one community participant or professional stakeholder aligned with all other factors.

**Conclusion:**

Results support the view that the public has a relatively sophisticated understanding of causes of health inequalities and help challenge assumptions held by policy actors that lay members of the public do not recognise and understand more upstream ways to respond to health inequalities.

**Patient or Public Contribution:**

The public contributed to the design of the Q study. Surveys and interviews with community participants informed the development of the statement set and the statement set was also piloted with community participants and finalised based on feedback.

## 1 Background

All UK political parties recognise the need to tackle health inequalities [1, 2]. Yet, rather than focusing on ‘upstream’ causes of poor health, policy often focuses on modifying individuals' behaviours [3, 4]. The importance of engaging with the public as a way to influence policy decisions, particularly towards more radical policy change, is recognised by policy actors [5]. An established body of work has explored public perceptions on causes of health inequalities [6, 7, 8]. This identifies a shift in how the public conceives of poor health from a focus on individual behaviour to recognising social factors as drivers of health. But we know much less about public perceptions on how to tackle health inequalities in the UK, with only a handful of academic studies [9, 10, 11, 12].

A Q methodology study based in Glasgow, Scotland, featuring community participants with low incomes and professional stakeholders found three shared views on solutions to health inequalities focusing on empowering communities, individual choices and redistribution [11]. Community participants, with experience of socioeconomic disadvantage, did not align with the view on redistribution and professional stakeholders did not share the view on individual choices. In contrast, a study combining a nationally representative survey and citizens' juries, found public support for improving living and working conditions to tackle health inequalities [12]. A different Q methodology study, based on a remote‐rural island community in Scotland, also found support for redistributing resources to improve rural health alongside other views around ways to empower communities and curb negative health behaviours [10]. Finally, a qualitative study exploring views of younger participants on potential solutions to health inequalities described the importance of living and working standards as the most likely way to tackle health inequalities [9].

This handful of studies highlight the diverse ways in which different subsets of the public, such as those with experience of socioeconomic disadvantage, rural islanders and young people, conceptualise responses to health inequalities. The existence of plural views is perhaps unsurprising given the lack of evidence on mechanisms for tackling health inequalities [13, 14, 15] and because people think differently about things. Enhancing this evidence base is also possible through engagement with the public, who can offer new perspectives, insights and evidence in this regard [5]. While the importance of ‘lived experience’ is increasingly recognised in policymaking [16, 17], so far, no study has focused on how individuals with very poor health conceive of solutions to health inequalities as well as their causes. Policy actors believe the public, in general, hold individualised, behavioural views of health inequalities [5, 18] which often align with neoliberal and medicalised policy responses [19, 20]. Currently, we do not know if individualised, behavioural views have been internalised by those who are among the most vulnerable in society or if a social determinants of health view is supported. Identifying and articulating the perspectives of this ‘public’ also offers opportunities to explore differences regarding how health inequalities are conceived of within this ‘public’ and between other ‘publics’, such as professional stakeholders providing different forms of support and other community participants with different experiences of socioeconomic disadvantage and health based in different geographic locations. In this paper, we respond to these two gaps. We build on a very small literature to strengthen the understanding of public(s) views on actions to address health inequalities, as well as their causes, by honing in on those groups who experience both poor health and low income. Specifically, we do this via a Q methodological study with low‐income participants, living in communities in London and experiencing one or multiple long‐term conditions, alongside a sample of other (professional) stakeholders.

## 2 Methods

### 2.1 Q Methodology

Q methodology examines subjectivity [21]. Subsequent to rank‐ordering statements of opinion onto a quasi‐normal shaped grid (a card‐sort), by‐person factor analysis is used to establish patterns of similarity based on correlating the card‐sorts, and from which shared views (or factors) emerge. We utilise the same statement set as the Glasgow Q methodology study [11] to elicit perspectives on causes of, and solutions to, health inequalities with community participants and professional stakeholders. The methods are described in full elsewhere (see McHugh et al. [11]), including how community participants contributed to the design of the Q study.

### 2.2 Data Collection

Based on their view, participants ranked 34 statements on ‘Causes’ and 39 on ‘Solutions’ (see Tables 1 and 2) onto corresponding quasi‐normal shaped grids. Grid scales were from −4 to +4 (‘Causes’) and from −5 to +5 (‘Solutions’), with all statements prefixed by ‘Health is worse in low‐income communities because…’ (‘Causes’) or ‘Health could be improved in low‐income communities by…’ (‘Solutions’). Each participant card‐sorted ‘Causes’ first, followed by ‘Solutions’. A post‐sort qualitative interview was audio‐recorded and transcribed verbatim, exploring respondents' general views on the topic in question and reasons for the placement of statements reflecting their most strongly held views. This information was used to aid selection of factor solutions and factor interpretations.

**Table 1:** London: ‘Causes’ statement set and ranks.

S. No.	Health is worse in low‐income communities because… Statement	F1	F2	F3
1	…people are unable to access space or places to meet others	−1*	2	0
2	…people don't have good support networks	0*	3*	−1*
3	…people feel like they are excluded from the rest of society	0	0	2
4	…there isn't enough community spirit	−3*	0*	−2*
5	…of low levels of education	3*	−1*	0*
6	…of unpredictable finances	4*	0	1
7	…there is a lack of insight into what these communities need	1	3*	0
8	…people see others in society with status symbols like expensive cars which make them feel bad about their own situation because they can't afford them	−2	−1	0
9	…of the stress of making hard decisions like ‘do we eat?’ or ‘do we heat?’	1	1	3*
10	…people don't get to experience the outdoors like being in the mountains, forests or by the sea	0	−2*	0
11	…there is a lack of good quality, affordable housing	4	2	2
12	…there aren't things for young people to do in their community	1*	4*	−1*
13	…of how the welfare system works	2*	0	1
14	…people struggle to get access to services that are available	2	0	1
*15*	*…many people don't have jobs that are secure meaningful or that give them a sense of purpose*	*3*	*3*	*4*
16	…people feel a sense of hopelessness from not being in control	0	1	4*
*17*	*…people lack the ability to look after themselves*	−*2*	−*1*	−*2*
18	…people can struggle with complicated family life, sexual, emotional or physical abuse	2	2	1
*19*	*…the culture of the community means people don't have ambitions or goals*	−*3*	−*3*	−*3*
20	…people are labelled, stereotyped and talked down to, they are not treated as individuals	−1*	1	2
21	…the views of these communities aren't taken into account	1	1	0*
*22*	*…it is difficult to leave an area to start a new life*	−*1*	*0*	−*1*
23	…the people in these communities can't cope with unexpected events or costs	1	−1*	3*
24	…these communities tend to be dirty, polluted or in poor condition	2*	−3*	−1*
25	…people don't have a way to travel, can't afford car or public transport	−1	−1	2*
26	…having less money increases the cost of things people need like electricity or loans	0	1	3
*27*	*…of poor parenting*	−*3*	−*2*	−*2*
28	…people in these communities don't follow health advice	−1	−2	−2
29	…people don't feel safe where they are living	0	−2*	−1
*30*	*…there is a culture of dependency and laziness in these communities*	−*4*	−*4*	−*4*
31	…people in these communities don't take responsibility for their own health	−2	−4	−4
32	…governments don't invest in these communities	3	4	1*
33	…people have too many children	−4	−3	−3
34	…people focus on short‐term pleasures rather than thinking about the future	−2*	2*	−3*

* Indicates distinguishing statements at *p* < 0.01. Italics indicate consensus statements nonsignificant at *p* > 0.05.

**Table 2:** London: ‘Solutions’ statement set and ranks.

S.No.	Health could be improved in low‐income communities by… Statements	F1	F2	F3
*1*	*…making free childcare available and accessible*	*2*	*1*	*1*
2	…spending more on the NHS	3*	−1	−1
3	…providing better support to rehabilitate prisoners, ex‐offenders or people who have had addiction problems	2*	−1*	−3*
4	…supporting industries, companies or sectors that can provide ‘good work’	1*	−2	−3
*5*	*…investing in community activities and groups which give people something to do*	*0*	*1*	*0*
6	…focusing on how we better support vulnerable individuals like young men, young mums or older people	4	4	1*
*7*	*…increasing the availability of, and access to, social care services in these areas*	*2*	*1*	*2*
8	…helping people to develop their strengths	−1*	3	4
9	…helping people to make relationships with others so that they have someone to look out for them or to turn to when things get hard	0	2	4
10	…making it possible for people to access affordable, flexible loans when they need them	−1*	0*	−4*
11	…increasing the tax on things that are bad for people like alcohol, sugary food and drink or fatty foods	−3*	−4*	0*
12	…improving the quality of housing for people on low incomes	5*	3	3
13	…making sure that people have enough money each month to pay their basic needs like rent, food, clothing, heat for their home	5*	0	3
*14*	*…cutting welfare benefits*	−*5*	−*5*	−*5*
15	…making sure that everyone who wants a job can get a job	1*	−2	−1
16	…legalising drugs	−2*	−4	−4
17	…making sure that everyone in society has similar opportunities	4*	−2*	0*
18	…by raising the taxes that people pay in a fair way	0*	−3*	2*
19	…providing ways for people to talk about and deal with mental health issues	3	2	0*
20	…better educating children about health from a young age	1*	3	4
21	…making sure communities have a say in any decisions that will affect them	2	5	3
22	…providing services that help people to organise their money like financial advice	−1	2	1
23	…providing safe ways for individuals to own their home, a car, things like that without getting into debt that they can't repay	−2	−1	−4*
24	…encouraging children to have goals and to have confidence to meet them	0*	2*	5*
25	…having more health campaigns	−3*	0*	−1*
26	…people taking responsibility for themselves	−4*	4	2
27	…finding more ways for people from different groups or different communities in society to mix together	−1	−1	1
28	…improving the availability and price of public transport	−1	1*	−1
29	…helping communities to own land, buildings or other assets in their community	−2	0*	−2
30	…reducing the price of things that are good for you like healthy food	0*	−1*	5*
31	providing coaching sessions for good parenting	−2*	1	2
*32*	*…denying healthcare to people who are responsible for their own condition like smokers or fat people*	−*5*	−*4*	−*5*
33	…stopping benefit payments to those spending their money on things that are bad for own health	−4	−3	−2*
34	…why should we do anything? if people want to make bad choices for their health then let them	−4	−3	−3
35	…improving the environment of the community so that it is easier for people to be active outside	1	4*	0
36	…by controlling what shops in these communities can sell	−3	−5	−2*
37	…making more funding available for good primary healthcare, such as GP surgeries or community pharmacists, in these areas	4	5	1*
38	…these communities deciding what needs to be done to improve health and then doing it	3*	0*	−2*
39	…preventing payday or doorstep lenders from taking advantage of vulnerable individuals	1	−2	−1

* Indicates distinguishing statements at *p* < 0.01. Italics indicate consensus statements nonsignificant at *p* > 0.05.

Participants were targeted from two main groups in London: community participants and professional stakeholders. The community participant group comprised working‐age individuals living on low incomes with one or more long‐term condition(s) living in the Boroughs of Lambeth and Southwark, London, England. Around half the population of these Boroughs belongs to black, Asian and other ethnic groups. Many are born outside the UK and experience poor health, with one in five (i.e. 140,000) residents living with at least one long‐term health condition, such as diabetes, chronic kidney disease or heart disease and depression, and over 19,000 residents live with three or more [22]. To facilitate access to this seldom‐heard population, we recruited a subsample of individuals who were participating in the ‘FinWell London’ research project having been recruited through a range of community organisations [23]. The materials were translated into Spanish by native Spanish speakers in the research team for participants who could not speak English. For the professional stakeholder group, we purposively targeted and contacted individuals local to London with expertise around different aspects – such as public health, community development, financial, legal and housing services – related to social determinants of health.

### 2.3 Analysis

Standard Q analysis was undertaken to define factors for both ‘Causes’ and ‘Solutions’ in this London study before exploring the relationship between these results and with the Glasgow Q study [11].

#### 2.3.1 London Q Analysis

‘Causes’ and ‘Solutions’ datasets were analysed separately using the Q software package KADE [24] to identify underlying latent structures. Varimax rotation followed centroid factor extraction. Factors contained at least two *defining* card‐sorts: (i) whereby a statistically significant (*p* < 0.05) factor loading (correlation) exists between card‐sort and factor and (ii) where such cards‐sorts must have greater association with one factor than all other factors combined (i.e. accounting for the majority of common variance). Factors also had to be interpretable and coherent according to idealised (composite) card‐sorts and post‐sort interview data.

Holistic descriptions of each factor (or shared viewpoint) are then produced from the selected factor solution by referring to the placement of statements on idealised card‐sorts (see Tables 1 and 2). This comprises a distinctive ranking of the original statement set for each factor based on a weighted average of defining card‐sorts; a statement with a ranking of +4, for example, indicates it is in the most agree column of the 'Causes' grid for the corresponding factor. Interpretation utilises different types of statements: *characterising* (i.e. statements placed at extremities of the grid about which participants feel most strongly); *distinguishing* (i.e. statements sorted differently in one factor compared to all others, assessed by statistical significance); and *consensus* (i.e. statements between pairs of factors with nonsignificant differences). Additionally, post‐sort interview data from those participants with *defining* card‐sorts were thematically analysed to aid interpretation. These interpretations form the centrepiece of the findings.

#### 2.3.2 London Q Study: ‘Causes’ Versus ‘Solutions’

Within sample analysis explores the relationship between the ‘Causes’ and ‘Solutions’ factor solutions in the London study. Factor descriptions were examined qualitatively and also quantitatively by correlating the factor loadings for pairs of factors. The latter explores, for example, if respondents who aligned with London ‘Causes’ Factor 1 (CL_1_) also aligned with London ‘Solutions’ Factor 1 (SL_1_).

#### 2.3.3 London Versus Glasgow

Between sample analysis focuses on relationships between the results of the London and Glasgow Q studies. This was also explored qualitatively through examination of factor descriptions, and quantitatively by estimating correlations for pairs of idealised card‐sorts from each factor solution. The latter indicates how similar the factors are from each study by exploring, for example, the relationship between CL_1_ and each of the Glasgow ‘Causes’ factors, for example, CG_1_, CG_2_ and CG_3_.

## 3 Results

Data were collected in London between September 2019 and February 2020. Card‐sorts for both ‘Causes’ and ‘Solutions’ were completed by 40 respondents (20 community participants and 20 professional stakeholders – see Tables 3 and 4). Almost all of our 20 community participants were female, ranging from 27 to 67 years of age with over half having a non‐British background. Everyone in the sample was managing at least one long‐term health condition with 13 community participants managing three or more, 9 were registered as disabled, 18 were receiving means‐tested benefits and only two were in formal employment. Data were collected from four community participants using translated materials. The 20 professional stakeholders were from five broad categories: academia, healthcare, the Third Sector, Government and financial services.

**Table 3:** Summary characteristics of full respondent sample (*n* = 40) and respondents defining the factor.

	Summary characteristics		Total	‘Causes’ Defining^a^ Sorts			‘Solutions’ Defining^a^ Sorts		
				CL_1_ (*n* = 11)	CL_2_ (*n* = 5)	CL_3_ (*n* = 11)	SL_1_ (*n* = 22)	SL_2_ (*n* = 8)	SL_3_ (*n* = 5)
**Community Participants (*n*** = **20)**	Age	18–30	3	0	0	1	0	0	3
		31–50	8	2	0	2	3	2	1
		51–64	7	1	4	2	4	2	0
		65+	2	0	1	0	1	0	1
	Gender	Female	19	2	5	5	7	4	5
		Male	1	1	0	0	1	0	0
	Ethnic Background	British	8	1	2	2	3	2	2
		non‐British	12	2	3	3	5	2	3
	No. of Long‐Term Health Conditions^b^	1	2	1	0	0	1	0	1
		2	4	0	1	1	2	1	1
		3+	13	2	4	4	4	3	3
	Registered as Disabled^b^	Yes	9	1	4	2	5	1	2
		No	10	2	1	3	2	3	3
	Welfare Benefits (Means‐Tested)^b^	Yes	18	3	5	5	7	4	4
		No	1	0	0	0	0	0	1
	Full/Part‐Time Employed	Yes	2	0	1	0	2	0	0
		No	18	3	4	5	6	4	5
**Professional Stakeholders (*n*** = **20)**	Expertise	Academic	4	2	0	1	2	0	1
		Healthcare	2	1	0	0	1	0	1
		Third Sector	9	4	0	3	8	1	0
		Government	1	0	0	1	1	0	0
		Financial Services	4	1	0	1	1	1	0

^a^ Defining card‐sorts have a significant association and majority common variance.

^b^ Missing data from one participant.

**Table 4:** Respondents' background, expertise and factor loadings.^a^

ID	Background/expertise	**‘**Causes**’**			**‘**Solutions**’**		
		CL_1_	CL_2_	CL_3_	SL_1_	SL_2_	SL_3_
**PS17**	Healthcare – Public Health Doctor	**0.79X**	0.22	0.27	**0.52X**	0.13	0.23
**CP12**	1 LTC, not disabled, on benefits, unemployed, British	**0.74X**	0.28	0.17	**0.69X**	0.23	0.25
**PS08**	Third Sector – Advice	**0.72X**	0.24	**0.52**	**0.60X**	**0.39**	−0.02
**PS20**	Academic – Public Health Policy	**0.72X**	−0.02	−0.09	**0.47**	**0.51**	0.30
**PS04**	Third Sector – Charity Public Policy Researcher	**0.65X**	0.16	**0.50**	**0.74X**	0.12	0.22
**PS15**	Third Sector – Charity Public Health Policy Researcher	**0.63X**	0.24	**0.56**	**0.73X**	0.13	0.13
**PS09**	Third Sector – Community Worker	**0.59X**	**0.37**	0.30	**0.75X**	0.15	0.12
**CP07**	3 + LTC, disabled, on benefits, unemployed, non‐British	**0.58X**	0.20	0.27	**0.61X**	**0.48**	0.17
**PS01**	Academic – Public Health	**0.58X**	0.06	**0.49**	**0.66X**	**0.37**	−0.02
**CP10**	3 + LTC, not disabled, on benefits, unemployed, non‐British	**0.56X**	0.09	−0.10	**0.35**	**0.48**	**0.45**
**PS02**	Financial Services – Social Investment	**0.42X**	0.15	0.12	0.19	**0.73X**	0.05
**CP02**	3 + LTC, not disabled, on benefits, unemployed, British	0.21	**0.65X**	0.12	**0.33**	**0.70X**	0.24
**CP19**	3 + LTC, disabled, on benefits, employed, non‐British	0.28	**0.60X**	0.18	**0.41X**	0.20	0.02
**CP05**	3 + LTC, disabled, on benefits, unemployed, non‐British	0.25	**0.53X**	0.24	**0.61X**	0.29	−0.04
**CP11**	3 + LTC, disabled, on benefits, unemployed, British	0.25	**0.39X**	0.11	**0.63X**	**0.33**	0.07
**CP17**	2 LTC, disabled, on benefits, unemployed, British	0.08	**0.37X**	0.24	**0.51X**	0.20	0.22
**PS13**	Third Sector – Charity Social Policy	**0.46**	0.12	**0.73X**	0.19	**0.56X**	0.29
**PS11**	Academic – Philosophy and Public Policy	**0.45**	0.14	**0.71X**	**0.73X**	**0.37**	0.00
**CP09**	3 + LTC, not disabled, on benefits, unemployed, non‐British	−0.05	0.31	**0.68X**	0.30	−0.12	**0.32**
**PS05**	Third Sector – Legal Aid	**0.57**	0.19	**0.63X**	**0.83X**	0.08	0.04
**PS03**	Financial Services – Money Advice	0.29	**0.49**	**0.60X**	**0.41**	**0.46**	**0.47**
**PS14**	Government – Politician	**0.34**	0.32	**0.60X**	**0.80X**	**0.38**	−0.00
**CP18**	2 LTC, not disabled, on benefits, unemployed, non‐British	0.21	**0.45**	**0.59X**	**0.71X**	0.22	0.20
**PS18**	Third Sector – Social and Community Psychiatry	0.31	0.05	**0.58X**	**0.71X**	−0.07	0.30
**CP16**	3 + LTC, disabled, on benefits, unemployed, non‐British	0.06	**0.36**	**0.58X**	**0.33**	**0.60X**	0.15
**CP06**	3 + LTC, disabled, on benefits, unemployed, British	0.16	0.16	**0.41X**	−0.07	0.07	**0.38X**
**CP14**	3 + LTC, not disabled, on benefits, unemployed, British	0.04	0.19	**0.41X**	0.05	**0.54X**	0.13
**PS06**	Financial Services – Community Development	**0.64**	0.29	**0.58**	**0.85X**	**0.36**	0.10
**PS19**	Healthcare – General Practitioner Doctor	**0.54**	0.29	**0.51**	**0.45**	0.24	**0.62X**
**PS10**	Third Sector – Welfare Rights	**0.47**	**0.40**	**0.58**	**0.73X**	0.14	**0.34**
**PS12**	Financial Services – Loans and Money Advice	**0.47**	**0.54**	0.27	**0.77X**	**0.34**	0.15
**PS16**	Third Sector – Migrants' and Women's Rights	**0.42**	0.24	**0.46**	**0.55X**	**0.38**	0.08
**CP15**	Employed, non‐British^b^	0.27	0.12	0.01	**0.40X**	−0.14	0.17
**CP13**	1 LTC, not disabled, on benefits, unemployed, non‐British	0.18	0.29	0.14	0.26	0.31	**0.54X**
**CP08**	3 + LTC, not disabled, on benefits, unemployed, non‐British	0.15	0.17	0.16	0.04	0.07	**0.48X**
**CP04**	3 + LTC, not disabled, not on benefits, unemployed, non‐British	0.07	0.02	**−0.49**	0.22	**0.55X**	**0.39**
**PS07**	Academic – Public Health	0.04	0.20	0.07	0.05	**0.38**	**0.50X**
**CP01**	3 + LTC, disabled, on benefits, unemployed, non‐British	−0.02	0.27	−0.00	0.29	**0.35**	**0.44**
**CP20**	2 LTC, not disabled, on benefits, unemployed, British	−0.08	−0.30	0.32	0.01	**0.56X**	**0.33**
**CP03**	2 LTC, disabled, on benefits, unemployed, non‐British	**−0.46**	**−0.50**	**−0.34**	**0.41**	**0.50X**	−0.17
**% EV**		19	10	18	28	14	8

*Note:* Significant factor loadings are shown in bold.

Abbreviations: CP = community participants, EV = explained variance, LTC = long‐term condition, PS = professional stakeholders.

^a^ The table is ordered by factor loadings on ‘Causes’. The factor loadings of defining card‐sorts are indicated with an X. These loadings meet the following two criteria: (i) the loading is statistically significant (*p* < 0.05). The significance level is calculated as 1.96 × (SE). SE represents standard error that is defined as 1/√*N* where *N* is the number of statements in the statement set. For ‘Causes’, 1.96 × (SE) = 1.96 (1/√34) = 0.34. For ‘Solutions’, 1.96 × (SE) = 1.96 (1/√39) = 0.31. (ii) the square of the loading for a factor is larger than the sum of the square loadings for all other factors (i.e. majority common variance).

^b^ Missing background data for this participant.

Three‐factor solutions were identified for each of ‘Causes’ and ‘Solutions’ – see Table 4. These were supported statistically, yielding interpretable accounts consistent with qualitative data. Tables 1 and 2 show, for each factor, the idealised card‐sorts based on the position of each statement on a grid. Whether positively or negatively, all 40 card‐sorts are associated to some degree with the three accounts within each factor solution (see Table 4). From Tables 3 and 4, it can be seen that, using a significance level of *p* < 0.05 and majority common variance, ‘Causes’ factors are defined by 11, 5 and 11 card‐sorts respectively (identified by an ‘X’ in Table 4), whilst ‘Solutions’ factors are defined by 22, 8 and 5 card‐sorts. For example, PS17 defines ‘Causes’ F1 (see Table 4). Card‐sorts with significant loadings on more than one factor and without majority common variance are called ‘mixed loaders’; an example of this is PS06 in ‘Causes’. Card‐sorts which do not load significantly on any factor are called ‘null loaders’; an example of which is CP08 in ‘Causes’. No professional stakeholders defined account C‐2 while at least one community participant or professional stakeholder defined all other accounts.

### 3.1 Factor Descriptions

Narratives of factors are based on the idealised card‐sort for each factor (see Tables 1 and 2) combined with the post‐sort interviews of defining card sorters (see Table 4). Brief overviews of ‘Causes’ and ‘Solutions’ are presented in Box 1 and are derived from interpretations of the idealised card‐sorts in Tables 1 and 2 and post‐sort interviews that are described in full in Appendix 1.

Box 1 **Brief overview of the london factor descriptions (see appendix 1 for the full factor descriptions).**

**London: ‘Causes’**

***CL***_***1***_ ***: Systemic inequality and poverty***

Systemic inequality and poverty impact people's individual choices, living environments and opportunities.

***CL***_***2***_ ***: Ignored and marginalised communities***

Management of difficult situations and leading healthy lives are made harder by lack of investment in low‐income communities and local service cutbacks.

***CL***_***3***_ ***: Precariousness, chronic stress and hopelessness***

Lack of money and financial vulnerability each lead to absence of hope and chronic stress, resulting in the worse health experienced by those in low‐income communities.

**London: ‘Solutions’**

***SL***_***1***_ ***: Meeting basic needs and providing opportunities to thrive***

Enabling people to pay for heating, clothing, food and rent and live in good quality housing, can provide a platform to create societies in which everyone has equal opportunities to thrive.

***SL***_***2***_ ***: Empowering individuals to take control***

Improving individual decision‐making capabilities and giving them agency to control their own environment will empower individuals to take responsibility for their own future, as opposed to imposing top‐down social policies from the Government.

***SL***_***3***_ ***: Supporting healthy choices***

Better support systems, in terms of networks, services and meeting basic needs, throughout a person's life are required to help people choose healthier lifestyles.

### 3.2 Exploring the Relationship Between ‘Causes’ and ‘Solutions’ in London

Pearson correlations between factor loadings of pairs of London ‘Causes’ and ‘Solutions’ viewpoints are in Table 5. All ‘Causes’ viewpoints have a positive association with SL_1_; this is statistically significant for CL_1_ and CL_3_. This is not unexpected given that SL_1_ dominates with regard to the percentage of explained variance, has card‐sorts with higher factor loadings and more defining card‐sorts (see Table 4). Qualitatively there is also alignment. All the ‘Causes’ viewpoints focus on different aspects of structural and community issues that would be improved by reducing the precariousness of individuals' lives and improving the opportunities available to individuals in low‐income communities. All other ‘Causes’ viewpoints have a negative or negligible association with SL_2_ and SL_3_. Again, there is qualitative alignment. SL_2_ and SL_3_ focus on structural issues to a lesser degree with more of an onus on individual decision‐making, choices and the autonomy of the individual.

**Table 5:** Correlations between factor loadings of London ‘Causes’ and ‘Solutions’.

	SL_1_: Meeting basic needs and providing opportunities to thrive	SL_2_: Empowering individuals to take control	SL_3_: Supporting healthy choices
CL_1_: Systemic inequality and poverty	0.58***	−0.12	−0.09
CL_2_: Ignored and marginalised communities	0.28	−0.13	0.05
CL_3_: Precariousness, chronic stress and hopelessness	0.36**	−0.26	−0.12

***1% significance level, **5% significance level.

### 3.3 How Similar Are the Viewpoints Between London and Glasgow

A brief summary of the Glasgow ‘Causes’ and ‘Solutions’ Q study findings [11] are reported in Box 2. Tables 6 and 7, respectively, then show correlations between the London and Glasgow factor solutions based on the similarity between the idealised card‐sorts calculated for each factor. These quantitative findings are qualitatively supported by the factor descriptions.

Box 2 **Brief overview of the glasgow factor descriptions (see McHugh et al. [11] for the full factor descriptions).**

**Glasgow: ‘Causes’**

***CG***_***1***_ ***: Unfair society***

Health inequalities are viewed as structurally determined, not only through housing, the welfare system, employment and the wider economy, but also the politicised environment in which these structures operate.

***CG***_***2***_ ***: Dependent workless and lazy***

Individuals' lost motivation and abilities to look after themselves due to over‐reliance on the state have resulted in health being worse in low‐income communities.

***CG***_***3***_ ***: Intergenerational hardships***

Health is worse in low‐income communities due to intergenerational family situations which are complex and have worsened because government investment and policies are poorly targeted.

**Glasgow: ‘Solutions’**

***SG***_***1***_ ***: Empower communities***

Health could be improved in low‐income communities by devolution of power to communities to decide on actions.

***SG***_***2***_ ***: Paternalism***

Putting in place supportive frameworks will enable those in low‐income communities to improve their health through better choices.

***SG***_***3***_ ***: Redistribution***

Structural changes are needed that address health inequalities by targeting the distribution of income, wealth and power in society.

**Table 6:** ‘Causes’: Correlations between London and Glasgow idealised card‐sorts.

	CG_1_: Unfair society	CG_2_: Dependent, workless and lazy	CG_3_: Intergenerational hardships
CL_1_: Systemic inequality and poverty	0.76***	0.03	0.48***
CL_2_: Ignored and marginalised communities	0.63***	−0.04	0.17
CL_3_: Precariousness, chronic stress and hopelessness	0.83***	0.14	0.48***

***1% significance level, **5% significance level.

**Table 7:** ‘Solutions’: Correlations between London and Glasgow idealised card‐sorts.

	SG_1_: Empower communities	SG_2_: Paternalism	SG_3_: Redistribution
SL_1_: Meeting basic needs and providing opportunities to thrive	0.66***	0.48***	0.71***
SL_2_: Empowering individuals to take control	0.61***	0.57***	0.19
SL_3_: Supporting healthy choices	0.57***	0.59***	0.30

***1% significance level, **5% significance level.

The Glasgow ‘Causes’ Q study identified three factors (see Box 2): ‘Unfair society’ (CG_1_); ‘Dependent workless and lazy’ (CG_2_) and ‘Intergenerational hardships’ (CG_3_). Only community participants define CG_2_. CG_1_ and CG_3_ are defined by both community participants and professional stakeholders. Table 6 highlights the (high) statistically significant correlation between CG_1_ (‘Unfair society’) and all three London ‘Causes’ viewpoints and similarly so between CG_3_ (‘Intergenerational hardships’) and CL_1_ (‘Systemic inequality and poverty’) and CL_3_ (‘Precariousness, chronic stress and hopelessness’). There is no relationship between CG_2_ (‘Dependent, workless and lazy’) and any of the London ‘Causes’ viewpoints.

The Glasgow ‘Solutions’ Q study also identified three factors (see Box 2): ‘Empower communities’ (SG_1_); ‘Paternalism’ (SG_2_) and ‘Redistribution’ (SG_3_). Only community participants define SG_2_, only professional stakeholders define SG_3_ and SG_1_ is defined by both community participants and professional stakeholders. Table 7 shows that SG_1_ (‘Empower communities’) and SG_2_ (‘Paternalism’) and have high, statistically significant, correlations with all three London ‘Solutions’ viewpoints and that SG_3_ (‘Redistribution’) has a very high, statistically significant, correlation with SL_1_ (‘Meeting basic needs and providing opportunities to thrive’).

Overall, there are broad similarities across the London and Glasgow findings. The differences are starker with regard to ‘Causes’, in particular CG_2_ which was not found in the London study. Additionally, while community participants in London recognised structural responses are required to tackle health inequalities, the need for more redistributive policies did not align with the views of community participants in Glasgow.

## 4 Discussion

This study presents evidence of plural views around causes of, and solutions to, health inequalities among a sample of community participants, living with low incomes and poor health, and professional stakeholders from London. We discuss these findings, consider them in relation to the existing literature and highlight new areas of research to further develop this evidence base.

### 4.1 ‘Causes’ of Health Inequalities

The three identified shared perspectives from the London Q study present nuanced accounts of different structural, community and psychosocial factors that can result in health inequalities. CL_1_ emphasises the unpredictability of finances and low levels of education. CL_2_ stresses the importance of community factors, such as having things for young people to do, lack of support networks and community spaces. Lastly, CL_3_ is the most explicit in linking financial vulnerability, in terms of not having enough money, to psychosocial factors around stress and lack of control. Across the three accounts, there is broad agreement that precarious employment and lack of good quality affordable housing are key issues. Similarly, all accounts in the London Q study reject blaming individuals and communities for their poorer health and recognise such views have negative consequences for health. Interestingly, while CL_1_ and CL_3_ bring together the views of community participants and professional stakeholders; only community participants share the views represented by CL_2_. Thus, for a subset of community participants, community infrastructure and environment is very much to the fore in how the causes of health inequalities are understood.

These accounts align with reviews on lay conceptions of poor health and a recent study exploring public awareness of health inequalities which find the public, in general, are well informed about the causes of health inequalities and expected life expectancies of different societal groups [7, 8, 25]. Our findings are also in line with established epidemiological evidence that exists on UK socioeconomic inequalities in health [4]. This suggests a sophisticated understanding of the causes of health inequalities among a sample of community participants.

In comparison to the Glasgow Q study [11] one result stands out. The viewpoint – ‘Dependent, workless and lazy’ (CG_2_) – from the Glasgow Q study, defined only by community participants, is not found amongst this sample. This view emphasises individual responsibility and behaviour. The reasons for this are unclear and warrant further investigation. One explanation could be the difference in the characteristics of community participants between these two studies. While both samples comprise individuals with experience of socioeconomic disadvantage, all London community participants have at least one long‐term health condition and almost half the sample are registered as disabled making this group amongst the most vulnerable in the country; out of 25 community participants in the Glasgow Q study, five had one long‐term health condition and only three had multiple long‐term health conditions. Additionally, the London sample is more ethnically diverse with over half the sample identifying as non‐British. There is evidence from the three London ‘Causes’ viewpoints that those, particularly from minority groups, living with low incomes experience negative stereotyping and difficulties accessing entitlements. However, this sample may not have internalised, to the same extent, the experiences of the poverty‐based stigma and individual responsibility discourses, such as ‘undeservingness’, as those living on low incomes in Scotland and may resist ‘othering’ due to their experience with ill health [26, 27].

### 4.2 ‘Solutions’ to Health Inequalities

There is more variation in the three shared perspectives identified from the London Q study on how to improve the health of those living in low‐income communities. SL_1_ focuses on the root causes of ill health, emphasising the importance of meeting basic needs, prevention activities and equality of opportunity. SL_2_ centres on improving individuals' capacity to control their own future and autonomy. Finally, SL_3_, similar to SL_2_, describes wanting to help individuals make better choices but the emphasis is on providing individuals with a framework and the means to do so by introducing supportive measures from an early age, and like SL_1_, making policy changes so it is easier to make healthy choices. Across the accounts, there is importance placed on free, accessible childcare, and increasing access to social care. All accounts strongly reject cutting welfare benefits or denying healthcare to people who are responsible for their own condition.

Variations of SL_1_, SL_2_ and SL_3_ are apparent in the wider literature. SL_1_ is similar to findings from the four other UK studies exploring public views on how to address health inequalities which find support for prioritising structural responses and improving living and working conditions [9, 10, 11, 12]. Taken together, these findings help challenge assumptions by policy actors that lay members of the public do not recognise and support more upstream ways to respond to health inequalities [5, 18]. While SL_2_ has a high association with the views ‘Empower communities’ (SG_1_) and ‘Paternalism’ (SG_2_) from the Glasgow Q study the emphasis is different. Unlike ‘Paternalism’, SL_2_ places more emphasis on developing the individual than introducing policies to alter living and working environments. The focus on empowerment makes SL_2_ more like ‘Empower communities’ (SG_1_) but differs as SL_2_ describes empowering individuals rather than communities. Beyond the UK, SL_2_ is similar to the findings from studies in Australia [28] and the USA [29] which focus on individual responsibility, particularly Lundell et al. [29] which also identifies some resistance to top‐down, state‐led efforts to change individual behaviour. To the best of our knowledge, the studies by Putland et al. [28] and Lundell et al. [29] are the only other studies globally to explore public perceptions on how to tackle health inequalities. Finally, SL_3_ is very similar to the views ‘Empower communities’ (SG_1_) and ‘Paternalism’ (SG_2_) from the Glasgow Q study with its focus on making it easier for individuals to make healthy choices by introducing supportive frameworks (‘Paternalism’) and covering basic needs (‘Empower communities’).

Community participants and professional stakeholders share all three accounts in the London study. This highlights that some community participants have an understanding that structural responses are required to address health inequalities. This differs from the Glasgow Q study where no community participants defined the view ‘Redistribution’ (SG_3_). Again, the reason for this difference is not clear and warrants further investigation. It could relate to a combination of the relatively small sample size typical of Q studies and the difference in characteristics, particularly in relation to health, of the two samples of community participants. Importantly, these results also highlight plural views exist among professional stakeholders suggesting that there is no one agreed way to tackle health inequalities.

#### 4.2.1 The Need for New Research on Public Views and Health Inequalities

Engaging the public and understanding views on solutions to health inequalities has the potential to generate new knowledge of how to address health inequalities in addition to gaining pubic acceptance for more transformative policies [11, 30]. Yet, globally, this is an under researched area. The findings of the London and Glasgow Q studies on causes of, and solutions to, health inequalities point to three avenues of further research.

First, developing Q‐based survey methods (see e.g., Mason et al. [31]) of the Glasgow and London Q studies. Q methodology is used to uncover the details on viewpoints and does not make claims about the representativeness of the accounts identified. However, the findings of Q studies can be used to develop related survey questions to explore the prevalence and distribution of the viewpoints identified, among a representative sample. Thus, providing new insight into the extent to which the general public holds individualised, behavioural views of health inequalities, for example, and their support for structural responses. Second, combining Q methodology (and/or Q‐based survey methods) with deliberative methods. A before and after Q study along with a deliberative activity [32, 33] could provide insight into whether, how and why certain views about health inequalities (do not) change in relation to the reasoned exchange of views and the provision of balanced information. Alternatively, the results of a Q‐based survey could form part of the evidence discussed in a deliberative activity between experts, policy actors and/or the public. This would help to represent the views of the public more broadly within deliberative activities which are typically undertaken with relatively small samples. Third, developing stated preference‐based survey questions on potential policy solutions. A number of different policies could correspond to different aspects of the views identified. For example, meeting basic needs by introducing a Universal Basic Income or increasing the size of current welfare benefits, such as Universal Credit. Smith et al. [12] is the only study to utilise quantitative methods to explore views on solutions to health inequalities. However, respondents are not required to make trade‐offs so the value placed on different policies is unknown.

## 5 Limitations

The framing of statements could impact their ranking, particularly how community participants consider the causes of health inequalities [34]. However, in line with Watts and Stenner [21] we chose to retain statements in the naturalistic language they were expressed in during the development of the statement sets. The framing of individual statements is also less of a concern in Q methodology where the focus is on holistic interpretations of idealised card‐sorts rather than the interpretation of individual statements more typical in R methodology. It is also possible that individual views, as represented by individual card‐sorts, could change particularly following some new experience. However, as Q methodology is concerned with shared viewpoints (factors), this is less of an issue and these shared viewpoints are much less likely to change.

## 6 Conclusion

We find nuanced and plural accounts of the perceived causes of, and solutions to, the worse health of those living in low‐income communities from a Q study with community participants living with low incomes and poor health and professional stakeholders from London. Our results support the view that the public has a relatively sophisticated understanding of the causes of health inequalities and help challenge assumptions held by policy actors that lay members of the public do not recognise and understand more upstream ways to respond to health inequalities [5, 7, 18]. Despite the different views, our results point to areas of convergence amongst community participants and professional stakeholders related to, for example, precarious employment and lack of good quality affordable housing causing health inequalities and the importance of free, accessible childcare, and increasing access to social care as possible responses. This could act as a helpful starting point to develop the goodwill necessary for further, likely more contested, conversations on how to tackle health inequalities. Across the London and Glasgow Q studies, while there were broad similarities two findings warrant further investigation. The view identifying individual responsibility and behaviour as causing health inequalities was not found in the London Q study and community participants in London, unlike those in Glasgow, recognised the need for structural responses to address health inequalities. The detailed accounts on causes of, and solutions to, health inequalities provide the basis for developing new areas of research, particularly around quantitative methods, to measure the extent to which these views are held in society and the value the general public attach to different policy solutions.

## Author Contributions

**Neil McHugh:** conceptualization, data curation, formal analysis, funding acquisition, methodology, project administration, supervision, validation, writing–original draft. **Rachel Baker:** conceptualization, funding acquisition, methodology, validation, writing–review and editing. **Cam Donaldson:** conceptualization, funding acquisition, methodology, validation, writing–review and editing. **Ahalya Bala:** formal analysis, investigation, methodology, project administration, validation, writing–review and editing. **Marta Mojarrieta:** investigation, methodology, validation, project administration, writing–review and editing. **Gregory White:** investigation, methodology, project administration, validation, writing–review and editing. **Olga Biosca:** conceptualization, funding acquisition, methodology, project administration, supervision, validation, writing–review and editing.

## Ethics Statement

Ethical approval was obtained from the Glasgow School for Business and Society Research Ethics Committee, Glasgow Caledonian University (Reference: GSBS‐EC‐014).

## Consent

Each participant provided informed consent before their participation. All participants provided informed consent for using their anonymised data for scientific publications.

## Conflicts of Interest

The authors declare no conflicts of interest.

## Supporting information

Supporting information.

## Data Availability

An anonymised data set containing the ranking of the statements by participants is available from the authors upon reasonable request.
