# Supplementary material for: Causes, Solutions and Health Inequalities: Comparing Perspectives of Professional Stakeholders and Community Participants Experiencing Low Income and Poor Health in London
Source: Health Expect. 2024 Dec 17;27(6):e70128. doi: 10.1111/hex.70128 (PMC11651171; doi:10.1111/hex.70128)
Supplement: Supplementary file 1 — Supporting information. [file HEX-27-e70128-s001.docx]

# **Appendix 1 – Full Factor Descriptions**

In the full factor descriptions reference is made to the position of statements in the idealised card-sort (see Table 1 and 2) and quotes from the post-sort interview are used to enrich both the interpretation and descriptions. The following notations are used: # indicates statement number and is shown with a number indicating its position on the Q-grid. For example, (#23, +3) indicates that statement 23 appears in position +3 for the factor in question.

# **‘Causes’ – Health is worse in low-income communities because . . .**

**Factor 1: Systemic inequality and poverty**

For this account, health is worse in low-income communities due to a combination of the “wider determinates of health, including inequalities in education, access to services and broader socio-economic variation” (PS17) which impacts individuals’ choices and their living environments.

People living in low-incomes communities tend not to enjoy the same opportunities in accessing “good quality education” (PS08) (#5, +3). This has “wide ranging implications across the full experience of someone’s life and over the entire course of it and is also intergenerational and persists” (PS17). Individuals are less likely to be “informed about their rights” (PS08), the services that are available to them (#14, +2) and what they are entitled to from the welfare system, in terms of means-tested, disability and in-work benefits (#13, +2). Failing to maximise income in this way makes it more difficult to live a healthy life particularly as finances will be more unpredictable (#6, +4). People with low-income are then faced with making stressful daily choices, such as do we eat or do we heat, and don’t have a buffer to deal with unexpected events or costs (#9, +1; #23, +1). This can result in individuals “doing jobs that they really aren’t well enough to do or that aren’t good for their health because they haven’t got any other choice” (PS04) (#15, +3).

The physical and family environments in which such individuals live are not conducive to good health. People living in low-income communities can struggle physically and mentally in poor quality housing which is more likely to be “cold, drafty, poorly insulated, with mould” (PS08) (#11, +4). Lack of government investment also means these communities are often in disrepair and that there isn’t anything for young people to do (#32, +3; #24, +2; #12, +1). To improve this situation and ensure future investment responds to what these communities actually need, the views of the community need to be taken into account during the decision-making process(#32, +3; #11, +4; #21, +1; #7, +1).

The daily grind of poverty in these communities also contributes to more dysfunctional and difficult family life. The focus on day-to-day survival (#6, +4; #15, +3; #13, +2) puts pressure on individuals and restricts their ability to contemplate or plan for the future (#34, -2): “poor people are planning until payday at the end of the week and rich people are planning for their grandchildren so that’s all about control I guess, if you can plan decades in advance, you have a great deal of control over your life” (PS20). People living on low-incomes are also more likely to have suffered some form of abuse (#18, +2). Thus, it isn’t through a want of trying or not having ambition to have a better life that their health is worse (#30, -4; #19, -3; #27, -3; #4, -3; #31, -2; #17, -2). Labelling people on low-income as lazy and dependent (#30, -4) or as having too many children (#33, -4) is prejudiced and ignorant. Doing so is often “used to discredit and marginalise people in these communities” (PS17). The reality is that people on low-incomes can’t “afford to be lazy” (CP12) and that “actually living and surviving and making the best of your circumstances is much harder in a low income community than it is in a high income community” (PS15). Consequently, people on low-income individuals have less time and means to make decisions that could be more conducive to better health.

**Factor 2: Ignored and marginalised communities**

This view sees health as worse in low-income communities because of a lack of investment in communities and cutbacks in existing services (#32, +4) which mean people lack the support to help them manage difficult situations and to lead healthy lives (#12, +4; #2, +3; #31, -4).

Cutbacks to key support services, such as those for mental health, mean services either no longer exist, operate with reduced hours or have much longer waiting lists (#32, +4; #2, +3). This makes Individuals’ ability to navigate a complex system to get help with “health, housing…, welfare … anything” (CP19) more difficult and services which do exist, such as the job centre, focus heavily on the stipulations of Universal Credit at the expense of empathy and people engagement. A lack of community space affects informal support networks which impacts on community spirit as there are less places available to meet and socialise (#2, +3; #1, +2; #4, 0). This can leave communities feeling “cold” and individuals “isolated” (CP05). Communities are not dirty or unsafe, but a lack of investment makes it difficult for communities to function well and can lead to difficulties (#32, +4; #24, -3; #29, -2). Young people in particular suffer as communities lack playgrounds, children’s centres, youth clubs and after school clubs or if the latter exist they are too expensive for parents to send their children to them (#12, +4; #32, +4; #1, +2). This means children and teenagers in particular spend more time on the streets where they are more likely to be “getting into violence and getting into drugs and getting into gangs” (CP11) which raises the risk of knife crime. Investment is prioritised in richer areas and the views and needs of low-income communities are ignored even when marches are organised to protest the closure of services and facilities for children (#7, +3; #21, +1).

The stereotype that a culture of dependency and laziness exists in low-income communities is heard on the news and perpetrated in society, particularly against minority groups (#30, -4; #33, -3; #20, +1). This effects individuals in daily life as people “look down at you before they even hear what you’ve got to say, so I think they’ve got this stereotype before they can even defend themselves” (CP19). Individuals in these communities want to take responsibility for themselves and have ambitions but don’t have the necessary means to do so (#31, -4; #19, -3; #28, -2; #17, -1). Lack of good quality affordable housing means people are forced to live in small and damp accommodation which can aggravate existing illnesses and complicated family lives mean individuals are more likely to suffer from depression (#11, +2; #18, +2). Individuals want to work and recognise how working can provide a sense of purpose. However, this is made difficult by the lack of meaningful jobs and being unable to afford to send children to nursery which means “if you want to go back to work and you can’t go back to work because you haven’t got the money to pay for that, you’re going to have to sit indoors and look after the kids yourself and you haven’t got the money so your health will deteriorate” (CP11) (#15, +3; #27, -2). A consequence of the situation individuals with low-incomes find themselves in is that they tend to “live for today and forget about tomorrow” (CP05), “as long as I’m alright and I’ve got food for today and I’ve got stuff that I need for today, blow tomorrow and blow next week…I’ve been like that since I was twelve years’ old” (CP11). This focus on short-term pleasures provides moments of relief and joy but can lead to worry and depression as it typically involves not contemplating the future and spending beyond one’s means (#34, +2).

**Factor 3: Precariousness, chronic stress and hopelessness**

The precarious financial situation of individuals in low-income communities causes “chronic stress” (PS11) and an “absence of hope” (PS13) which results in the worse health experienced by those in these communities (#16, +4; +9, +3).

Not having enough money makes it “harder for people to self-care” (PS05) and forces individuals to make stressful choices between how to feed their children and how to heat their home (#9, +3). Small unexpected things, such as a broken appliance or unexpected bill, can also be very stressful and very quickly “spiral out of control” (PS14) particularly where high cost credit is sought as money is needed quickly (#23, +3; #26, +3). Individuals do not have the “mental peace and calm” (PS13) in these situations in order to make better long-term decisions, such as seeking out a cheaper loan provider. The focus on the short-term is a necessity to get through until tomorrow (#34, -3).

Individuals experience a lack of control when it comes to employment and housing (#16, +4; #15, +4; #11, +2). An enjoyable job can offer a sense of inclusion and the potential of respite from problems in life - “if you’re a poor person and you’re at work, you’re not a poor person for the hours you’re at work, you’re a worker” (PS11) (#15, +4; #3, +2). However low-income individuals often have insecure employment through “zero-hour contracts” (PS13) which means weeks may go by when they don’t receive any hours which causes stress and makes it more difficult to cope with unexpected financial difficulties, to look themselves and others (#16, +4; #15, +4; #9, +3; #23, +3; #6, +1; #31, -4; #17, -2; #27, -2). Similarly, poor housing conditions which individuals aren’t able to change can often lead to physical and mental health problems through damp, mouldy and overcrowded living conditions (#16, +4; #2, +2).

Services and support provided through the welfare system and health sector are ostensibly there to help individuals in times of need (#13, +1; #14, +1). However, an individuals’ social class, health status and/or whether they are an immigrant can lead to stereotyping and discrimination – “systems that are supposedly there to help you in some ways but constantly treat you as someone who’s likely to be cheating the system, there’s a level of lack of human contact with the people who are meant to be providing services for you” (PS11) (#20, +2; #3, +2; #14, +1). This can be very dispiriting and damage people’s mental and physical health.

The idea that there is something in the culture of low-income communities or that they have “essential characteristics” (PS05) that lead to worse health is nonsense and a bigoted view (#30, -4; #31, -4; #19, -3; #33, -3; #27, -2; #17, -2; #28, -2). This form of stereotyping acts as an “oppression towards poor people” (CP18). For example, while poor parenting may exist it is not confined to low-income communities only (#27, -2; #20, +2; #3, +2) – “somebody could be a poor parent … because they don’t have any money and they can’t buy food. That doesn’t make them a poor parent, that makes them a desperate person” (CP18). Ultimately, it is a lot easier “to take responsibility for your own health if you are in a secure financial situation” (PS14).

**‘Solutions’ – Health could be improved in low-income communities by . . . . . .**

**Factor 1 - Meeting basic needs and providing opportunities to thrive**

Fundamental to improving the health of vulnerable individuals in low-income communities is making sure that their basic needs are met (#13, +5; #6, +4). This involves individuals being able to pay for rent, food, heating and clothing and ensuring that they live in good quality housing (#13, +5; #12, +5; #6, +4). Addressing these immediate concerns “would make a massive difference to people’s mental health and wellbeing, and to their overall health” and provide “financial security” (PS04) that enables them “to think about medium-term and longer-term issues” (PS12). This means avoiding any punitive policies that would reduce the amount of money individuals have, when “people get poorer, they're more likely to become unhealthy than healthy and… they're also going to be more vulnerable to all the stresses that are going to affect their mental health” **(**PS05) (#14, -5; #33, -4; #11, -3; #6, +4).

Once an individual’s basic needs are met they will have more “bandwidth and emotional capacity and resilience to deal with every other challenge in their life including their ability to manage their health, both physically and mental” (PS04). Key to this is the availabity of, and being able to access, good primary health, social care and mental health services in these communities (#37, +4; #19, +3; #3, +2; #7, +2). Importantly, these should be community-based programmes that are “geared towards prevention rather than cure” (PS05). The programmes and other community services should be designed to respond directly to issues identified by communities. For this to occur communities need to have a voice and some agency in what’s happening in their community (#38, +3; #21, +2; #36, -3); the mantra should be “work with communities rather than do things to communities” (PS14).

The ultimate goal should be creating a society where “everyone has the same opportunity to thrive” (PS04) (#17, +4). Such an approach recognises individuals have bad health behaviours, such as smoking and eating unhealthy food, due to “wider issues in their lives, which they're often not responsible for” (PS05) (#32, -5; #26, -4; #33, -4). Therefore, the focus should be on getting to the “root causes” (PS09) by providing free childcare and opportunities for people to gain employment and education (#17, +4; #6, +4; #3, +2; #4, +1; #15, +1; #20, +1). Rather than trying to get people to better manage their poor health through health campaigns or punishing them for their poor health by, for example, denying them access to health care (#32, -5; #14, -5; #33, -4; #34, -4; #25, -3).

**Factor 2 - Empowering individuals to take control**

Improving health in low-income communities requires that individuals are empowered to take responsibility for their own future (#21, +5; #26, +4; #8, +3). “Giving people agency” (PS02) allows them “to take control of their own environment” (PS02). When this happens, individuals are much more likely to use what is on offer in their community and in the most beneficial way. Individuals, particularly vulnerable individuals, such as young mums or older people, can gain control over their own life through the introduction of services which seek to improve individuals’ skill-sets and decision-making capability (#6, +4; #8, +3; #34, -3). For example, targeted services on specific issues, such as the provision of financial advice and mental health services and parental coaching sessions, can help individuals to manage challenging areas of their life (#22, +2; #19, +2; #31, +1). Similarly, pre-school childcare should teach children “about how to live well” (CP20) and adults should have the option of undertaking further education (#20, +3; #24, +2; #1, +1). However, individuals should not be punished “for their failure to take responsibility” (PS02), by cutting welfare benefits or denying people access to health care, as this would increase the pressure people are under and make them feel like they have less control of their life (#14, -5; #32, -4; #33, -3).

Creating a sense of agency in individuals is not achieved by everyone having the same opportunities, this will only give “people a sense of dependency” (PS02) (#17, -2; #26, +4). Individuals need to have choices and not have top-down solutions imposed on them through social policy as “all that happens is that people feel less and less empowered, because they feel the government controls more and more of their lives, less and less is within their control, they take less and less responsibility” (PS02). Policies which prohibit what people can spend their money on through banning or increasing the price of things, like unhealthy goods, are unlikely to change behaviour for the better and could lead to unwanted unintended consequences (#36, -5; #33, -2; #32, -4). Also polices which put more control in the hands of the Government, like increasing taxes that people pay should be avoided (#11, -4). The Government should focus on providing “the services that enable people to make themselves employable” (PS13) rather than forcing companies to provide jobs or choosing particular industries to support (#15, -2; #4, -2) – “It’s this sort of idea that Government somehow makes good choices and good decisions. I’m afraid there is ample evidence of the fact that, you know, an industrial policy can be completely cack-handed” (PS13).

Improving the environment of the community will give individuals more opportunity, and encourage them, to take control of their own life (#35, +4; #26, +4). Low-income individuals often live in areas with poor public transport and in housing which is poorly insulated and in disrepair which makes it difficult for them to be healthy (#12, +3; 28, +1). There should also be investment in good primary health and social care and mental health services in these communities (#37, +5; #19, +2; #7, +1). Ideally, these services would have a preventive focus and take the form of “wellbeing centres” (CP02). These centres could also then act as a hub for the community, a place where they could meet and network with others in the community and find out information on a range of things, particularly in relation to health and wellbeing (#35, +4; #8, +3; #9, +2; #5, +1). While individuals are “ultimately responsible for their own happiness, their own wellbeing” (PS13) improving their community in this way can help “to enable better behaviour” (PS13).

**Factor 3 – Supporting healthy choices**

Improving health comes down to individuals having better lifestyles – “increasing exercise and eating better diets and doing less of things that make you more ill (…) reducing cigarettes and alcohol” (PS19). For this to happen individuals need to make, and be supported to make, the right choices in life (#30, +5; #16, -4; #34, -3; #26, +2) – “a right choice is the healthy choice (…) bad choices are mostly related to unhealthy lifestyles” (PS07). This starts from childhood (#24, +5; #20, +4; #8, +4). Children need to be supported and encouraged to have goals and be confident to follow them – “if from when you are little you are told by your environment that you cannot, that you are limited because you don’t deserve it, limited by your background, because you don’t have money, and you believe that in your mind, that is going to limit you and make you think that you are useless. Create confident kids, deserving and able of everything, we are limitless” (CP13). An important part of this is instilling a “value set” (PS07) in people where they have “the competence and the skills” “to take some responsibility for how well they do in their life, whether that be their health, their job or other forms of success that people will value” (PS07).

To enable children and individuals to make healthy choices in their life the system should be set-up in a way that supports them. There are often good reasons why people make bad choices in terms of health behaviours; it’s to do with being on low income, not having a job, not having good housing and not having the time or money to access or afford healthy food (#30, +5; #32, -5; #14, -5; #13, +3; #12, +3; 33, -2). Ensuring that people have these basics can give people the time to think about their health and “do the things that they know would help” (PS19).

Better support systems, in terms of networks and services, around children and adults can also encourage and promote healthier lifestyles. Individuals being helped to make supportive relationships with others in, or outside, their own community will mean they have someone to look out for them or to turn to when things get hard (#9, +4; #27, +1). Parents and children would benefit from childcare being free, available and accessible for everybody and parents, as well as young adults and teenagers, should receive further education and coaching sessions about parenting (#20, +4; #31, +2; #1, +1; #6, +1). In addition to having good and more accessible primary health and social care services in the community, non-health services, such as financial advice, can help individuals to make decisions that improve their life money (#7, +2; #37, +1; #22, +1; #6, +1) – “That’s a skill, and we need a set of skills that will allow us to manage money, irrespective of how much money we have available to us” (PS07). Communities should have a say in how these supportive and mechanisms services are introduced and they should be paid for by fairly raising money through increased taxes that people pay (#21, +3; #18; +2).
